# Exploitation of a newly-identified entry pathway into the malaria parasite-infected erythrocyte to inhibit parasite egress

**DOI:** 10.1038/s41598-017-12258-x

**Published:** 2017-09-25

**Authors:** Svetlana Glushakova, Brad L. Busse, Matthias Garten, Josh R. Beck, Rick M. Fairhurst, Daniel E. Goldberg, Joshua Zimmerberg

**Affiliations:** 1 Section on Integrative Biophysics, Eunice Kennedy Shriver National Institute of Child Health and Human Development, National Institutes of Health, Bethesda, MD 20892 USA; 2 Laboratory of Malaria and Vector Research, National Institute of Allergy and Infectious Diseases; National Institutes of Health, Bethesda, MD 20892 USA; 3 Division of Infectious Diseases, Department of Medicine, Washington University, St. Louis, MO 63110 USA

## Abstract

While many parasites develop within host cells to avoid antibody responses and to utilize host cytoplasmic resources, elaborate egress processes have evolved to minimize the time between escaping and invading the next cell. In human erythrocytes, malaria parasites perforate their enclosing erythrocyte membrane shortly before egress. Here, we show that these pores clearly function as an entry pathway into infected erythrocytes for compounds that inhibit parasite egress. The natural glycosaminoglycan heparin surprisingly inhibited malaria parasite *egress*, trapping merozoites within infected erythrocytes. Labeled heparin neither bound to nor translocated through the intact erythrocyte membrane during parasite development, but fluxed into erythrocytes at the last minute of the parasite lifecycle. This short encounter was sufficient to significantly inhibit parasite egress and dispersion. Heparin blocks egress by interacting with both the surface of intra-erythrocytic merozoites and the inner aspect of erythrocyte membranes, preventing the rupture of infected erythrocytes but not parasitophorous vacuoles, and independently interfering with merozoite disaggregation. Since this action of heparin recapitulates that of neutralizing antibodies, membrane perforation presents a brief opportunity for a new strategy to inhibit parasite egress and replication.

## Introduction

To evade immune detection, intracellular apicomplexan parasites replicate within several layers of membranes^1,2^. However, this replication is only advantageous if parasite progeny can emerge successfully from host cells after breaking both the parasitophorous vacuolar membrane and the erythrocyte plasma membrane to invade new cells. The mechanics of malaria parasite egress from erythrocytes are still unresolved despite the growing knowledge of regulated enzymatic and signaling events involved in the egress mechanism^3^. Morphological transformations of the two parasite-enclosing membranes prior to parasite release were visualized in a limited number of studies^4–7^ in which it was suggested that vacuolar membrane rupture occurs prior to or simultaneous with the opening of the erythrocyte membrane. The functional proteins for vacuolar membrane rupture are not currently known. However, the erythrocyte cysteine protease calpain^8^ and the parasite surface protein MSP1^9^ have each been implicated in degradation of the erythrocyte cytoskeleton, a crucial step in parasite egress preparation leading to the opening of the host cell membrane. One of the final changes in the infected erythrocyte prior to egress is perforation of the erythrocyte membrane^10^. Perforation of the host cell membrane by Apicomplexan parasites was first demonstrated in *Toxoplasma*-infected cells^11^ and later was shown in *Plasmodium*-infected erythrocytes for both sexual and asexual forms^12–14^. *Toxoplasma* pore-forming protein 1 perforates both vacuolar and host plasma membranes while perforin-like protein 2 of *P. falciparum* is involved in perforation of the erythrocyte but not vacuolar membrane, and only in sexual forms of the parasite. Perforin-like protein 1 of *P. falciparum* was suggested to perforate both the parasitophorous vacuolar membrane and the erythrocyte membrane during the asexual cycle^15^, but a recent study demonstrated it is dispensable for blood-stage parasite growth^16^.

While investigating the inhibition of *P. falciparum* invasion of erythrocytes by heparin and other highly-charged sulfated glycosaminoglycans (GAGs)^17–20^, we noticed entry of these inhibitory compounds into infected cells through the membrane of erythrocytes. Entry could only occur at the very end of the replicative cycle, to inhibit parasite egress. This was a surprising observation, because in analogy to antiviral mechanisms^21^, it is postulated that heparin inhibits invasion *in vitro* by binding to merozoite surface proteins just prior to invasion, thus blocking subsequent merozoite binding to the erythrocyte surface^17^ (there is a clear non-specific loss of merozoite adhesion)^22^. Thus, perforation of the erythrocyte membrane just before parasite egress provides a novel entry pathway of high-molecular-weight hydrophilic compounds able to prevent parasite dissemination. Consistent with reports of delayed schizont rupture in the presence of several heparin-like compounds^19,23^, heparin exploits a natural stage in the parasite’s intra-erythrocytic developmental cycle (IDC), i.e. erythrocyte membrane perforation preceding membrane rupture during the last minute of the replicative cycle^12^. Our findings are the first indication of a technological application for the molecular-perforation stage of infected erythrocytes prior to egress.

## Results

### Heparin inhibits parasite egress ***in vitro***

**Figure 1:**
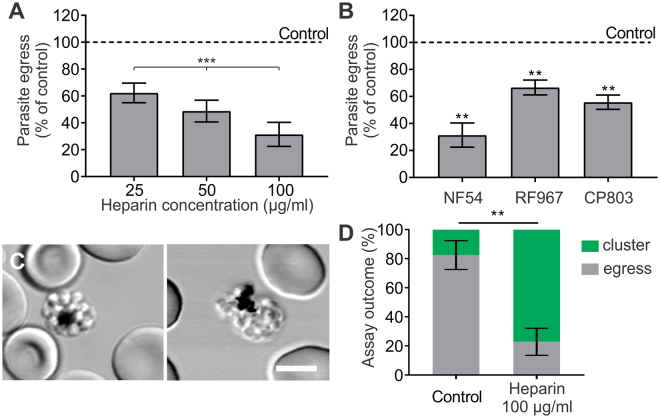
Heparin inhibits parasite egress *in vitro*. (**A**) *P. falciparum* NF54 schizonts were exposed to increasing concentrations of heparin for 60–90 min at 37 °C in environmental chambers that preserve sites of parasite egress. To stop the parasite IDC, chambers were cooled at 15 °C for 30 min and then examined by microscopy. Egress was quantified as the fraction of schizonts that released merozoites during the exposure time. A total of 800–1500 schizonts and egress sites were analyzed for each condition. Results are presented as mean ± SD. Heparin exerted a dose-dependent reduction in egress (one-way analysis of variance, ANOVA; p < 0.001). (**B**) NF54, RF967, and CP803 schizonts were exposed to 100 µg/ml of heparin for 60–90 min at 37 °C in environmental chambers, and egress assessed as in (**A**). A total of 1300–4400 schizonts and egress sites were analyzed for each condition. Results are presented as mean ± SD. Heparin significantly inhibited the egress of all three parasite strains (p < 0.01 for all, two-tailed one sample t-test, H_0_ = 100%). The effect of heparin on CP803 and RF967 egress was also dose-dependent (data not shown). (**C**) NF54 schizonts were exposed to 100 µg/ml of heparin for 60 min at 37 °C in environmental chambers, and imaged using laser-scanning confocal microscopy. Differential interference contrast (DIC) microscopy images show frequently-observed merozoite clusters. Scale bar = 5 µm. (**D**) Quantification of egress sites and clusters as a fraction of the sum of both assay outcomes, in control and heparin-treated cultures (100 µg/ml heparin, 2 h at 37 °C, n = 3, total number of counted infected cells and events = 900). The numbers of clusters in the heparin-treated culture is significantly increased compared to the control (P < 0.01, two-tailed t-test).

To assess heparin inhibition of parasite egress *in vitro*, we exposed *P. falciparum* schizonts to 25–100 µg/ml of heparin for 60–90 minutes at 37 °C. Using a quantitative egress assay^24^, we found that heparin had a reproducible, dose-dependent inhibitory effect on parasite egress from infected erythrocytes (Fig. 1A). Exposure to 100 µg/ml of heparin, which is known to strongly inhibit merozoite invasion of erythrocytes^17^, not only inhibited the egress of laboratory strain NF54 parasites to less than half of the control level but also inhibited the egress of two artemisinin-resistant *P. falciparum* clinical isolates, CP803 and RF967, up to 45% and 35%, respectively (Fig. 1B). These data suggest that heparin exerts a strain-transcendent inhibitory effect on parasite egress.

Live-cell microscopy of heparin-treated cultures revealed accumulation of morphologically-distorted infected cells composed of clustered merozoites mostly trapped within erythrocytes (Fig. 1C). Quantitative analysis of biological outcomes of the egress assay, i.e. counting the number of parasite egress sites and merozoite clusters in heparin-treated and control cultures, shows that (i) the sum of egress sites and clusters in heparin-treated cultures (98.0 ± 12.4% of the control, mean ± SD) were similar to those of the control (p = 0.83, two-tailed one sample t-test, H_0_ = 100%, n = 3), suggesting that both cultures normally progressed to the end of the cycle; and (ii) inhibition of egress in heparin-treated cultures is explained by significantly increased cluster formation (Fig. 1D). Together, our data suggest that heparin does not affect the last two hours of the parasite IDC, but interferes with the egress of mature merozoites.

### Mechanism of parasite egress inhibition by heparin

**Figure 2:**
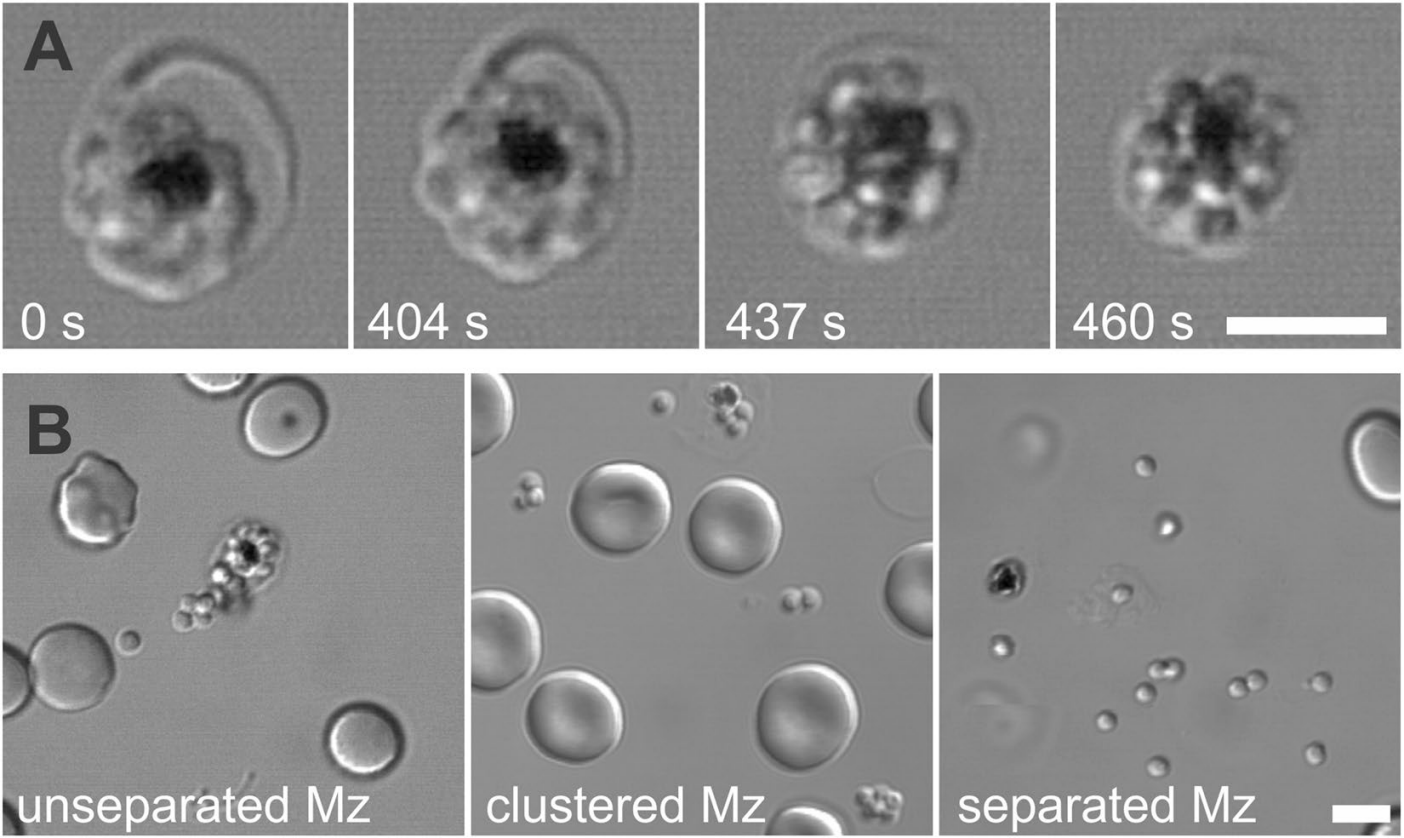
Schizonts exposed to heparin show different IDC outcomes. (**A**) Aborted parasite egress leading to formation of merozoite clusters within an erythrocyte. *P. falciparum* NF54 schizonts were exposed to 100 µg/ml of heparin at 37 °C in environmental chambers and parasite egress was recorded using laser-scanning confocal microscopy. DIC microscopy: selected frames from Supplemental Movie 1. Scale bars = 5 µm. (**B**) Inefficient merozoite dispersion. *P. falciparum* NF54 schizonts were exposed to 50 or 100 µg/ml of heparin for 60 min at 37 °C in environmental chambers and imaged using laser-scanning confocal microscopy. DIC microscopy images show examples of egress sites with unseparated, clustered, and separated merozoites (Mz). Note that only a limited number of single merozoites are available (left and middle images) to initiate a new cycle of erythrocyte invasion. Scale bar = 5 µm.

The final mechanical steps of egress include rupture of the parasitophorous vacuole, parasite dissociation within the schizont, and perforation and rupture of the erythrocyte membrane^5,10,12^. The scattered discharge of individual merozoites from the erythrocyte then completes the IDC^5^. To investigate the mechanism of egress inhibition by heparin, we first used time-lapse light microscopy to record parasite egress in medium containing 100 µg/ml of heparin and frequently observed blocked egresses (see Supplementary Movie 1 and Fig. 2A showing selected frames from this movie), leading to the formation of semi-dissociated merozoites, or merozoite clusters, retained within erythrocytes. The observed outcome mimics the static images presented in Fig. 1C. These data suggest that heparin interferes with the rupture of merozoite-enclosing membranes and likely affects merozoite dispersion prior to egress.

To further assess heparin’s effect on merozoite dispersion, we analyzed egress sites that are preserved in the chambers for microscopy and identified one prominent feature: egressed or partially-egressed merozoites were often in aggregates of varied sizes, indicating variable degrees of merozoite dispersion (Fig. 2B). 56.5 ± 11.4% (mean ± SD) of egress sites in cultures treated with 100 µg/ml of heparin (four independent experiments, n = 130) contained merozoite clusters of varied sizes in comparison to 17.7 ± 8.6% of sites in control cultures (three independent experiments, n = 97, p = 0.004, two-tailed t-test). The heparin-induced merozoite dispersion defect may account for part of heparin’s reported inhibition of parasite invasion, as merozoites that fail to detach from their siblings are expected to be invasion-incompetent.

**Figure 3:**
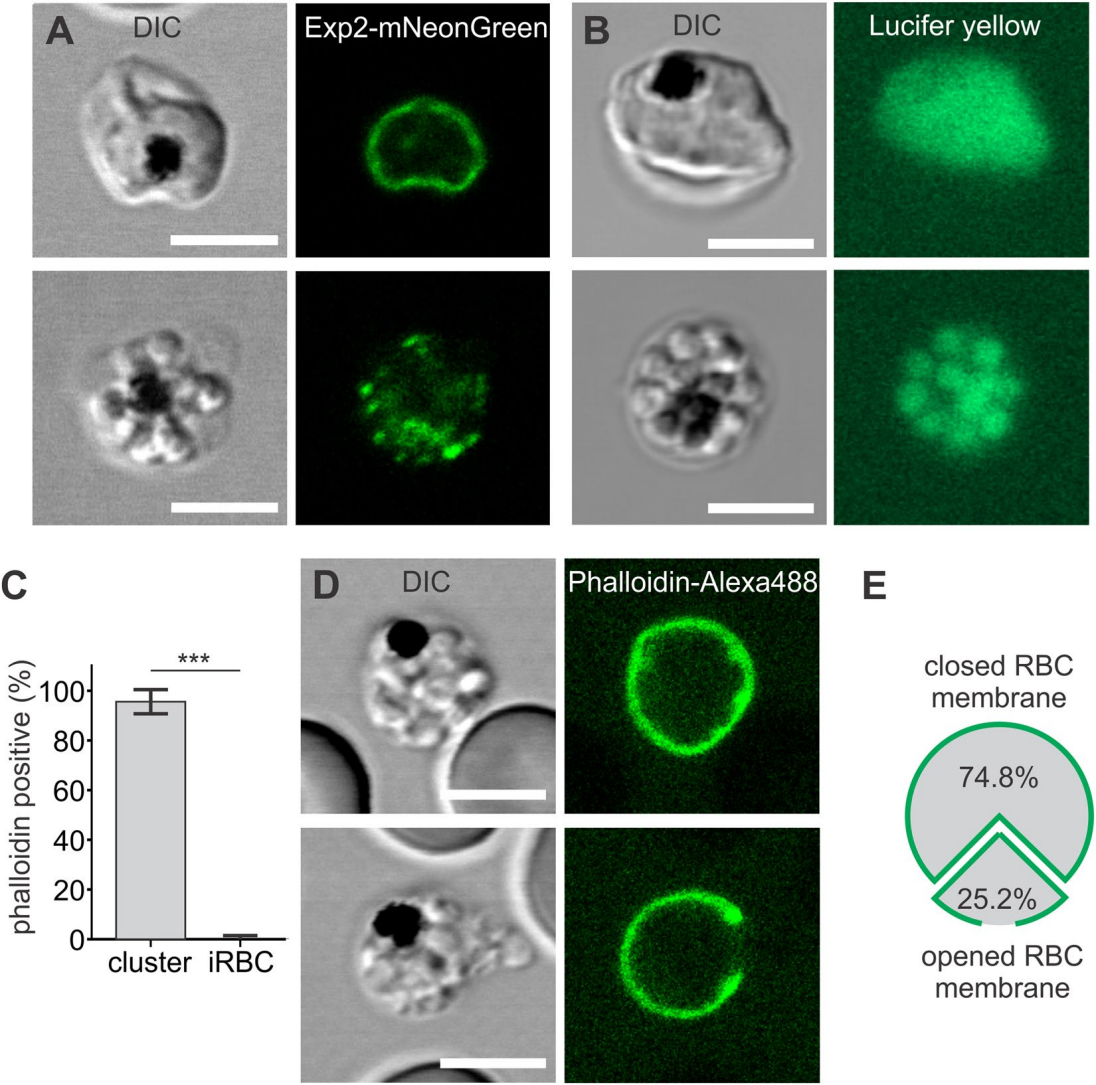
Heparin inhibits erythrocyte membrane rupture, but not PV membrane rupture. (**A**) To detect the status of PVM in infected cells, we used *P. falciparum* NF54 schizonts expressing EXP2-mNeonGreen as a PVM marker. Infected cells were imaged in control (upper panel) and heparin-treated cultures (lower panel) (100 µg/ml of heparin, 60 min at 37 °C). The majority of heparin-treated schizonts had a broken PVM, as gauged by fragmentation of the EXP2-mNeonGreen signal. (**B**) *P. falciparum* NF54 schizonts were incubated with Lucifer Yellow for 2.5 hours at 37 °C, washed, exposed to 100 µg/ml of heparin for 60 min at 37 °C, and imaged using laser-scanning confocal microscopy. DIC and fluorescence microscopy images show (upper panel) an immature schizont within an LY-filled PV, in which individual merozoites are obscured by accumulated LY in the PV, and (lower panel) intra-erythrocytic merozoite clusters surrounded by disintegrated, LY-leaking PVs, in which individual merozoites are clearly observed. Scale bars = 5 µm. (**C**) To detect the status of erythrocyte membrane in infected cells we used NF54 schizonts that were incubated in Alexa Fluor 488-phalloidin-supplemented medium with or without 100 µg/ml of heparin for 60 min at 37 °C and inspected cells using fluorescence microscopy. Almost all clusters in heparin-treated cultures were labeled with phalloidin-Alexa488 while normal infected erythrocytes were not (p < 0.001, paired two-tailed t-test). (**D**) The examples of phalloidin-Alexa 488-labeled clusters with apparently closed (upper panel) and opened membranes (lower panel). (**E**) Fraction of clusters with opened and closed membranes (n = 181).

To investigate the integrity of the parasitophorous vacuolar membrane (PVM) that encloses divided parasites, we generated an NF54 strain with a fluorescent mNeonGreen tag on the C-terminus of the endogenous EXP2 protein, a resident of the PVM^25^ (Fig. 3A, upper panel). EXP2-mNeonGreen infected cells were treated with 100 μg/ml heparin and inspected for PVM integrity using fluorescence microscopy. A broken PVM was observed in 85% of clustered merozoites (n = 60) in heparin-treated cultures (Fig. 3A, lower panel), in contrast to 5% of broken PVM in the apparently normal schizonts of control cultures (n = 64).

In a separate experiment to test the integrity of the PVM, we incubated infected cells with Lucifer Yellow CH (LY), a fluorescent marker for the PV space and merozoites (Fig. 3B, upper panel)^26,27^ and inspected dye distribution within infected cells in heparin-treated cultures. LY was not found around parasite clusters within infected cells (Fig. 3B, lower panel) but rather only in merozoites, presumably because LY leaked out of the PV upon rupture of the PVM. Together, these data suggest that most heparin-induced merozoite clusters harbored broken PVMs, and therefore that heparin does not interfere with PV disintegration at the end of the IDC.

To investigate whether heparin inhibits erythrocyte membrane rupture, we used Alexa Fluor 488-labeled phalloidin, which binds erythrocyte cytoskeletal F-actin^28^. Fluorescent phalloidin enters infected erythrocytes during parasite-induced perforation of the erythrocyte membrane, which precedes natural parasite egress^12,14^, and labels the inner erythrocyte membrane. When we exposed wild-type NF54 schizonts to Alexa Fluor 488-phalloidin and 100 µg/ml of heparin (three independent experiments) and analyzed them by fluorescence microscopy, we found that 95.6 ± 4.82% of inspected clusters (mean ± SD, n = 3, total number of inspected cells = 181) were surrounded with erythrocyte membrane permeable for fluorescent phalloidin, while only 0.55 ± 0.95% (mean ± SD, three experiments) of 181 morphologically normal schizonts in the same cultures were fluorescent (Fig. 3C). These data suggest that (i) heparin does not interfere with parasite-induced erythrocyte membrane perforation, (ii) clusters are enclosed in the erythrocyte membrane, and (iii) heparin was able to enter infected cells before parasite egress. Next, we evaluated macroscopic erythrocyte membrane integrity in 119 randomly-selected, phalloidin-labeled clusters in heparin-exposed cultures (cumulative data of two independent experiments). About 75% of the phalloidin-labeled erythrocytes contained merozoite clusters, enclosed in apparently intact erythrocyte membranes (Fig. 3D, upper panel, and Fig. 3E), while the rest of infected cells had macroscopic defects in the erythrocyte membrane (Fig. 3D, lower panel, and Fig. 3E). These data confirm that heparin interferes with erythrocyte membrane rupture or erythrocyte membrane shedding that could proceed once the membrane is broken.

Thus, heparin inhibits parasite egress and reinvasion by both blocking erythrocyte membrane rupture and impairing parasite dispersion at the end of the IDC.

### The mode of heparin interaction with infected erythrocytes and heparin targets

**Figure 4:**
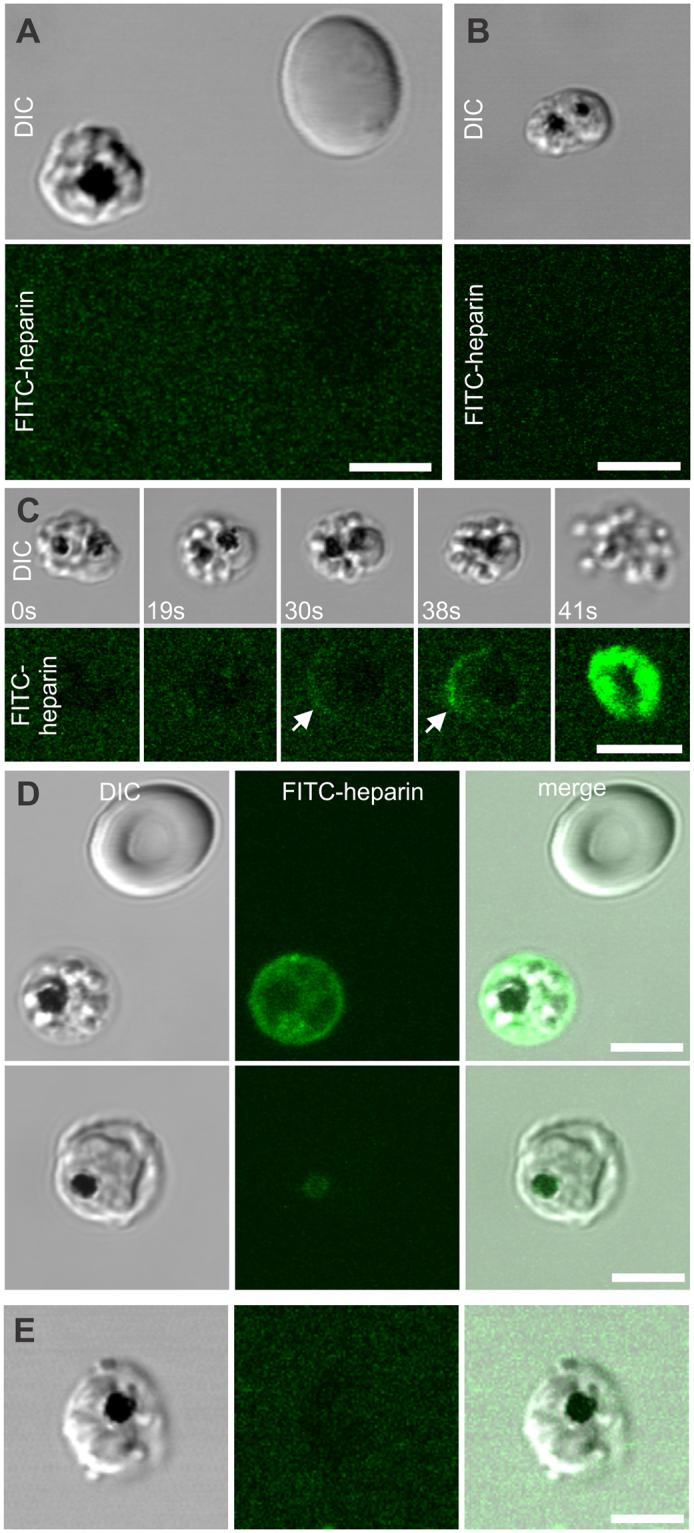
Heparin enters infected erythrocytes just prior to parasite egress. (**A**,**B**) *P. falciparum* NF54 schizonts were exposed to 10 (**A**) or 20 (**B**) µg/ml of FITC-heparin for 60 (**A**) or 120 min (**B**) at 37 °C in environmental chambers, and imaged using laser-scanning confocal microscopy. DIC and fluorescent microscopy images show schizonts a few seconds before parasite egress. (**C**) CP803 schizonts were incubated in 20 µg/ml of FITC-heparin for 60 min at 37 °C in environmental chambers, and imaged using laser-scanning confocal microscopy. DIC and fluorescent microscopy images from a live-cell recording of parasite egress (Supplemental Movie 2) are shown. (**D**) NF54 schizonts were incubated with 10 µg/ml of FITC-heparin and 10 µM of E-64 for 30 min at 37 °C, washed, placed in environmental chambers, and imaged using laser-scanning confocal microscopy. The upper panel shows an E-64-induced merozoite cluster filled with FITC-heparin that entered the perforated erythrocyte membrane, and an uninfected erythrocyte. The lower panel shows an immature schizont with an erythrocyte membrane that is still impermeable to FITC-heparin. (**E**) NF54 schizonts were incubated with 10 µg/ml of FITC-heparin and 4 µM of C2 for 30 min at 37 °C in environmental chambers, and imaged using laser-scanning confocal microscopy. C2-induced morphologically-abnormal schizonts were not labeled with FITC-Heparin (n = 60). Scale bars = 5 µm.

To investigate heparin’s interaction with infected erythrocytes and its molecular targets, we used a FITC-heparin conjugate and live-cell fluorescence microscopy. FITC-heparin was not found on the surface of uninfected or infected erythrocytes during 120 minutes of incubation (Fig. 4A,B) and did not accumulate within schizonts, which can acquire other labels^5,27^. Thus, intact membranes of uninfected and infected erythrocytes neither bind nor translocate these highly-charged polymerized heparin molecules, so the mechanism of egress inhibition is likely related to the very end of the cycle. To further explore the parasite-heparin interaction, we recorded parasite egress in the presence of FITC-heparin, and found that FITC-heparin conjugate enters pre-egressing schizonts just prior to erythrocyte membrane rupture (Fig. 4C shows selected frames from Supplementary Movie 2). This timing of FITC-heparin entry matches the previously-reported time for parasite-induced perforation of the erythrocyte membrane that precedes egress^12^. The pore size was apparently large enough for FITC-heparin influx, as was shown in experiments with the cysteine protease inhibitor E-64, which blocks parasite egress from erythrocytes but does not affect erythrocyte membrane perforation^10^. All inspected E-64-induced merozoite clusters contained FITC-heparin within erythrocytes (Fig. 4D, upper panel; compare with Fig. 4D, lower panel showing an immature schizont in the same culture with intact erythrocyte membrane not labeled with FITC-heparin). However, when a specific inhibitor of PfPKG, Compound 2 (C2)^29,30^, is used to entirely block parasite egress upstream of vacuolar and erythrocyte membrane perforation and rupture, morphologically-abnormal infected cells appear (63% of all infected cells, n = 200), which are different from the heparin-induced clusters and are not permeable for FITC-heparin (n = 60) (Fig. 4E). Thus, by investigating various stages of egress, both before erythrocyte membrane perforation (C2 treatment) and after this step (E-64 treatment), we have strengthened our conclusion that heparin can diffuse through parasite-modified erythrocyte membranes prior to egress.

**Figure 5:**
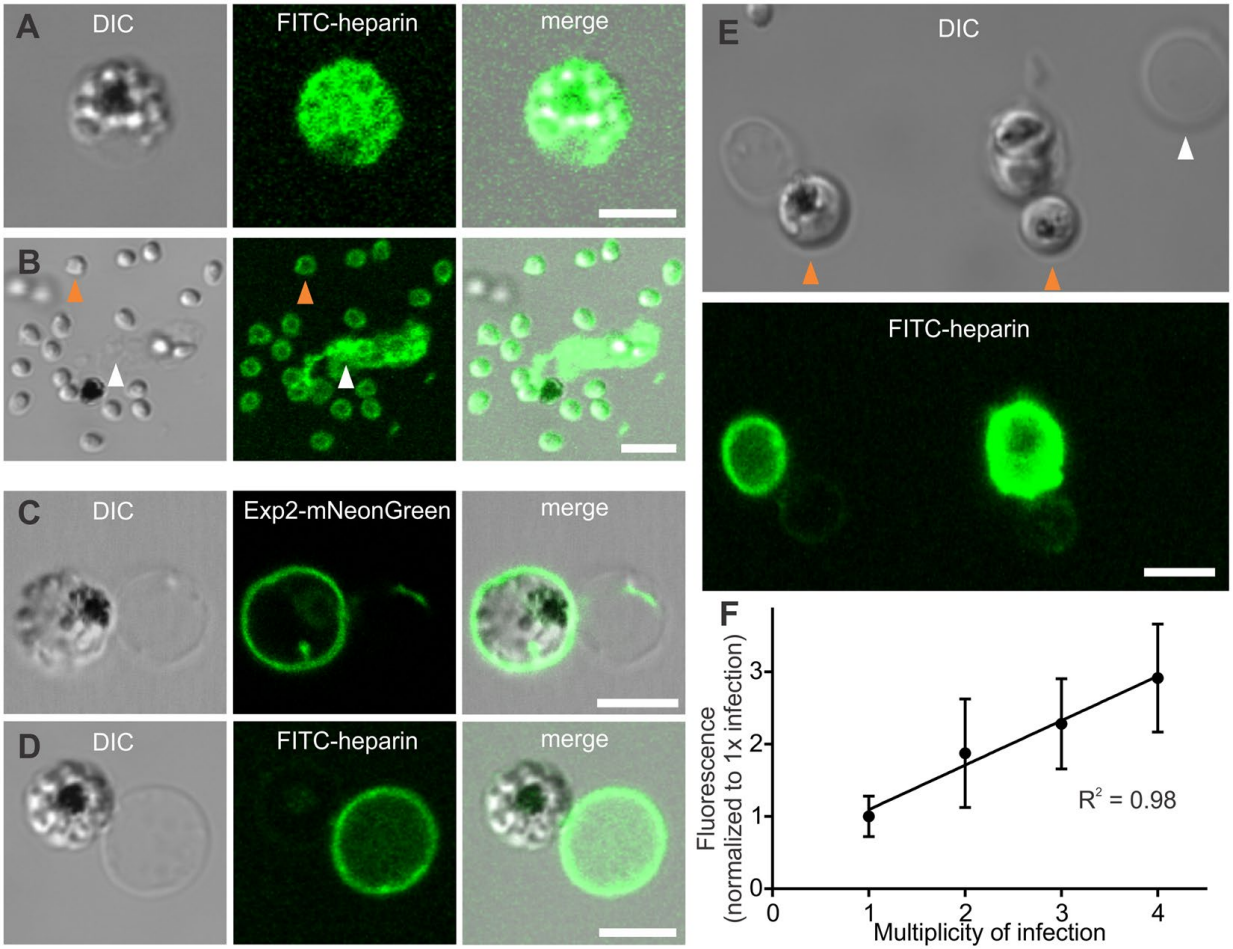
Heparin targets the inner erythrocyte membrane and the merozoite surface. (**A**,**B**) *P. falciparum* CP803 and NF54 schizonts were incubated with 5 (CP803) or 10 µg/ml (NF54) of FITC-heparin for 60 min at 37 °C in environmental chambers, and imaged using laser-scanning confocal microscopy. (**A**) NF54 merozoite cluster enclosed within a perforated erythrocyte. Note that both the merozoite and erythrocyte membranes are labeled with FITC-heparin. (**B**) CP803 parasite egress site containing labeled merozoites (orange arrowhead) and erythrocyte membrane remnants (white arrowhead). (**C**) NF54-EXP2-mNeonGreen schizonts were incubated in isotonic “high-potassium” buffer (140 mM KCl, 5 mM NaCl, 0.4 mM CaCl_2_, 0.4 mM MgCl_2,_ 25 mM HEPES, 4.5 mg/ml glucose, 0.5% Albumax II), and imaged using laser-scanning confocal microscopy. Due to the higher permeability of infected erythrocytes for potassium than sodium ions, many infected cells swelled, hemolyzed, and frequently extruded their vacuoles together with schizonts. An extruded schizont still retained within its PV judged by the presence of EXP2-mNeonGreen-labelled membrane around it. PV with schizont remains tethered to erythrocyte membrane. (**D**) A culture of NF54-infected cells was treated as described in (**C**) except that the “high-potassium” buffer was supplemented with 10 µg/ml of FITC-heparin. Note that FITC-heparin only labeled the erythrocyte ghost. PV membrane is impermeable to FITC-heparin. (**E**) Two RF967 parasites extruded from infected erythrocytes in the presence of FITC-heparin. Note the different intensity of erythrocyte membrane fluorescence in the single- versus the triple-infected erythrocyte. Both extruded parasites (orange arrowheads) are enclosed in PV membranes that are impermeable to FITC-heparin. Note that the non-infected erythrocyte ghost (white arrowhead) does not bind FITC-heparin. Scale bars = 5 µm. (**F**) NF54-infected cells were treated as described in (**D**) and imaged using laser-scanning confocal microscopy. The mean membrane pixel intensity of the individual erythrocyte ghosts of single-, double-, triple-, and quadruple-infected cells was assessed (n = 80) and taken as a data point. Data are presented as a normalized value to the value of the single-infected cells (mean ± SD). Line shows a result of the linear regression analysis, r^2^ = 0.98.

Two FITC-heparin targets were observed: the inner erythrocyte membrane just before egress or in the stalled parasite clusters, and the merozoite surface in clusters or egressed parasites (Fig. 5A,B). By observing the membranes of infected erythrocyte ghosts, which had extruded their PVM-enclosed parasites (we confirmed the presence of PVM around the extruded schizonts using EXP2-mNeonGreen-labeled, NF54-infected cells, Fig. 5C), we concluded that the FITC-heparin signal at the inner erythrocyte membrane (Fig. 5D) originated from a parasite source for two reasons: (i) uninfected erythrocyte ghosts do not bind FITC-heparin (n = 52, also see Fig. 5E, white arrowhead); and (ii) the intensity of erythrocyte membrane fluorescence is directly correlated (r^2^ = 0.98) with the multiplicity of erythrocyte infection (Fig. 5F, n = 80). These observations suggest that heparin targets are parasite-derived proteins, rather than infection-modified erythrocyte cytoskeletal proteins.

**Figure 6:**
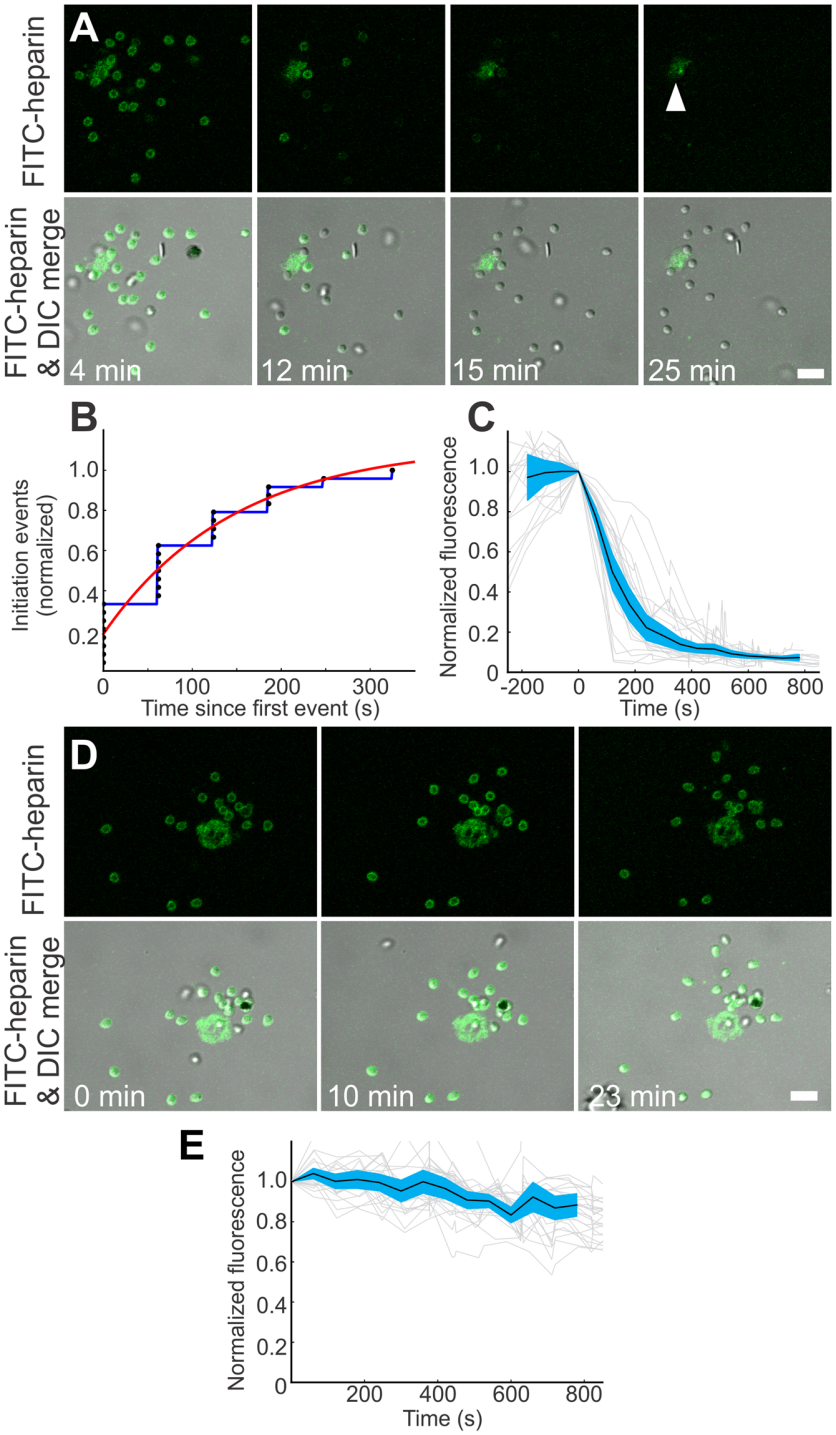
Ca^2+^-dependent shedding of heparin-binding target from the merozoite surface. (**A**–**C**) *P. falciparum* NF54 schizonts were incubated in 1 µg/ml of FITC-heparin for 60 min at 37 °C in environmental chambers, and imaged using laser-scanning confocal microscopy. (**A**) Selected frames from a representative egress site are shown (Supplementary Movie 3). Note that all in-focus merozoites show bright surface fluorescence at 4 min post egress, only two merozoites show weak surface fluorescence at 15 min post egress, and no merozoites show fluorescence at 20 min post egress; however, the erythrocyte membrane (white arrow) retains its fluorescence at 25 min post egress. (**B**) When the timing of merozoite fluorescence loss events are plotted together (t = 0, first fluorescence loss in each experiment), a logarithmic relationship (red) is revealed, suggesting that the initiation of fluorescence loss for each inspected merozoite has a stochastic component. (**C**) The mean time course of each merozoite’s fluorescence loss, aligned to the moment of initiation, shows that it occurs fast, within 2-3 min or roughly the same time scale (approximately 3 times slower) as invasion-induced shedding (within 1 min). Cyan band: 95% confidence interval. Light gray traces: individual merozoite intensities. (**D**,**E**) NF54 schizonts were incubated in 10 µg/ml of FITC-heparin and 5 mM EGTA for 60 min at 37 °C in environmental chambers, and imaged using laser-scanning confocal microscopy. Selected frames from a representative egress site are shown. Note that all in-focus merozoites show bright surface fluorescence for more than 20 min (**D**). Quantitative image analysis of individual merozoites in time-lapse recordings (every 60 seconds) shows that their rate of fluorescence loss after egressing from the same schizont was slow (**E**). Scale bars = 5 µm.

While the inner erythrocyte membrane showed a FITC-heparin fluorescence signal that was stable for several hours, merozoites showed a transient fluorescence signal that disappeared within 15 minutes after egress (Fig. 6A). Quantitative image analysis of individual merozoites released from the same schizont in time-lapse recordings (every 60 seconds) showed that fluorescence loss from the merozoite surface had a stochastic onset (Fig. 6B) and fast progression (Fig. 6C). Initiation of fluorescence loss was spread over a period of 205.2 ± 48.1 seconds (mean ± SEM) and characterized by an exponential with a time constant ± 95% confidence interval of 147.2 ± 81.5 seconds (Fig. 6B). Single-exponential decay equations fit to the fluorescence loss of parasites from the point of loss initiation (the end of the “shoulder” of fluorescence loss) had a time constant of 175.2 ± 72.6 seconds (mean ± SEM, four experiments with 3, 5, 7, and 5 individually-measured parasites in each experiment).

The high fluorescence intensity on merozoite surfaces indicates an abundance of FITC-heparin target. The transient nature of this signal suggests that heparin may bind proteins that are shed during merozoite invasion by the Ca^2+^-dependent subtilisin-type protease PfSUB2 and rhomboid proteases PfROM1 and PfROM4^31,32^. To discriminate between these, we investigated the Ca^2+^-dependence of heparin shedding *in vitro*. The drop in merozoite surface fluorescence was abolished when merozoites egressed into Ca^2+^-free medium (Fig. 6D), suggesting that the shedding of MSP1, the only known target of PfSUB2 on the merozoite surface that can bind heparin^33–36^, is causing the fluorescence loss. The enhanced stability of merozoite surface fluorescence in Ca^2+^-free medium enabled us to evaluate the photobleaching kinetics of FITC-heparin as a function of multiple scans. Quantitative image analysis revealed a slight decline in fluorescence over time (Fig. 6E), which can be ascribed to photobleaching. Thus, photobleaching was induced and measured by imaging the merozoites with frequent frame rates, and then quantified as a function of frame number (mean ± SEM frame constant, 35.7 ± 11.16 frames, n = 18 merozoites from two experiments). Knowing the rate of observed fluorescence decay in Ca^2+^-containing medium (one frame per minute), we calculated a time constant of decay due to photobleaching of 2140 seconds (~36 minutes), 12-fold longer than that observed above. This result excludes photobleaching of FITC as a significant factor in the disappearance of fluorescence from the merozoite surface in Ca^2+^-containing medium (Fig. 6C). The observed asynchronous nature of FITC-heparin shedding likely reflects a stochastic interaction between merozoite and putative shedding-inducing stimuli, such as receptors on erythrocyte membrane fragments at egress sites. After initiation, shedding proceeded at the same rate in most merozoites, supporting the possibility that it is an enzymatic reaction. Together, these data suggest that MSP1 is the most likely merozoite surface protein to bind heparin in our experiments.

**Figure 7:**
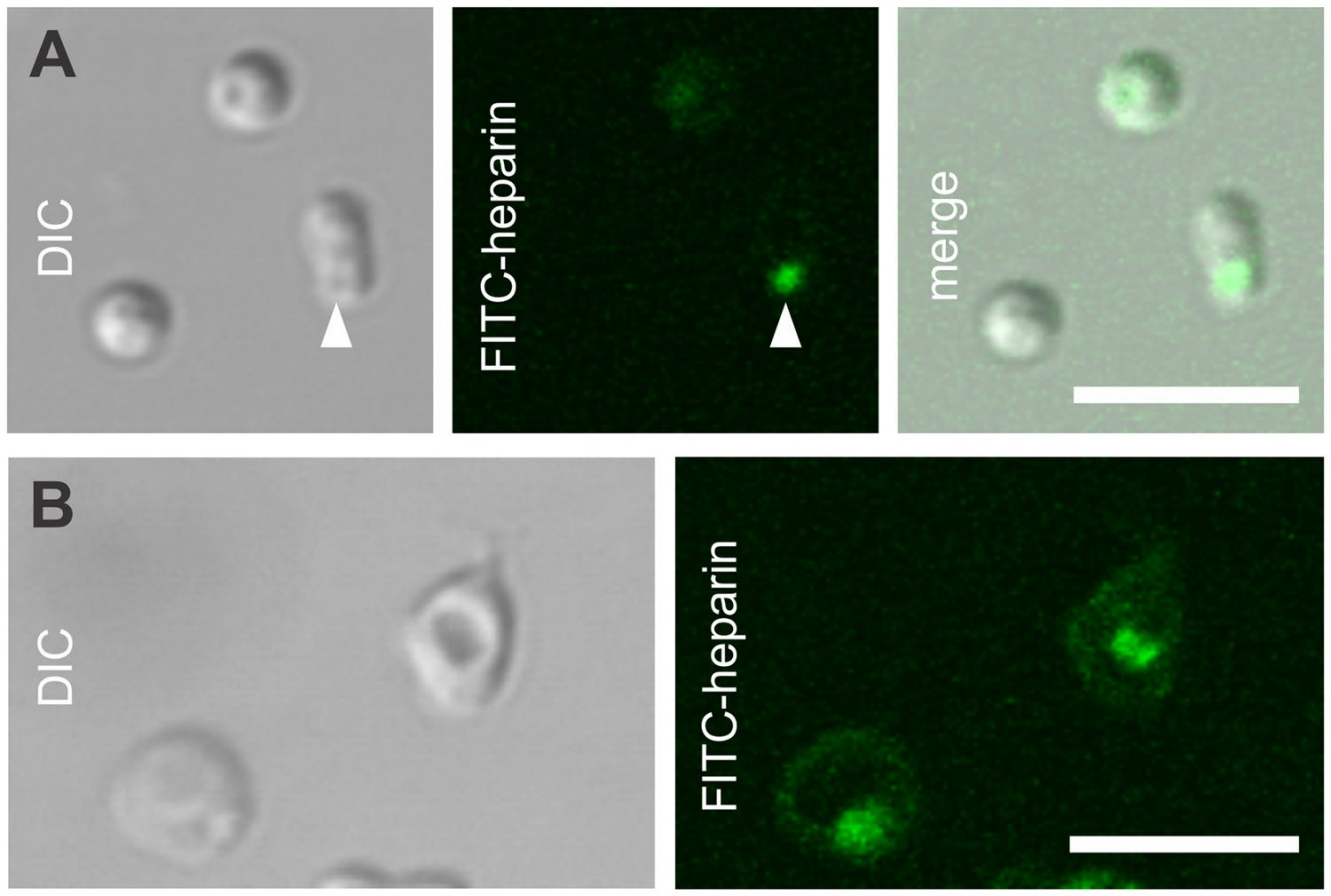
Heparin binds material extruded from the apical prominence of merozoites. (**A**,**B**) *P. falciparum* NF54 schizonts were exposed to 1 µg/ml of FITC-heparin for 60 min at 37 °C in environmental chambers, and imaged using laser-scanning confocal microscopy. (**A**) In Ca^2+^-containing medium, heparin is shed from the merozoite surface but also binds material extruded from the apical prominence (arrow) of merozoites. (**B**) In EGTA-containing medium, heparin remains bound to the merozoite surface but does not prevent the extrusion of heparin-binding material from the apical prominence. Scale bars = 5 µm.

A third heparin target was detected as the sudden appearance of a strong punctate fluorescence signal at the merozoite apical prominence (visualized by DIC microscopy) following shedding of heparin from the merozoite surface, i.e. not immediately after egress (Fig. 7A, Supplementary Movie 3), which then persisted for a prolonged period. The sequential nature of the shedding of heparin-binding merozoite surface proteins and appearance of apical fluorescence signal seem to represent two independent steps of the invasion process. Blocking the shedding of heparin from the merozoite surface in Ca^2+^-free medium did not affect the timely appearance of apical fluorescence signal on merozoites (Fig. 7B). The extrusion of apical material somewhat mimics the events of natural merozoite invasion *in vitro* ^37,38^ and may be involved in the reported inhibition of parasite invasion by heparin^17^, but not in Ca^2+^-free medium^39,40^.

## Discussion

Our data show that the highly-charged natural polymer heparin inhibits *P. falciparum* egress *in vitro*, exploiting a previously-unrecognized mode of interaction with infected erythrocytes from within, entering cells through parasite-derived pores in the erythrocyte membrane prior to parasite egress. The following heparin-cell interactions show the topology of proteins that are critically involved in the egress mechanism: surface proteins of merozoites and the inner aspect of erythrocyte membranes. The mechanics of egress inhibition includes inhibition of erythrocyte membrane rupture and prevention of merozoite dispersion. The fast kinetics of heparin binding to its targets and high intensity of FITC-heparin signal at the binding sites suggest that heparin has a high binding affinity for abundant parasite proteins. One of them is likely MSP1, a major merozoite surface protein that was recently implicated in parasite egress^9^. It was suggested that MSP1 interacts with spectrin, a component of the erythrocyte cytoskeleton, to disrupt the erythrocyte membrane and release merozoites. MSP1 is the most likely heparin target on the merozoite surface because it is a major merozoite surface protein and the only known heparin-binding protein to be shed from the merozoite surface by the Ca^2+^-dependent protease PfSUB2 after parasite egress^34,35,41^. Heparin-MSP1 binding may also explain poor merozoite dispersion. The heparin-induced merozoite clusters we observed appear to be the same as those induced by human antibodies specific for merozoite surface proteins^42^. The binding of polyvalent molecules such as heparin and antibodies to the merozoite surface may impair merozoite dispersion by effectively creating an adhesive between adjacent merozoites, preventing the formation of singular merozoites that are responsible for initiating a new round of erythrocyte invasion and parasite replication. Heparin binding to the inner erythrocyte membrane, which we suggest prevents erythrocyte membrane rupture and thus egress, could involve one or more parasite proteins known both to interact with components of the erythrocyte membrane and to bind heparin^36^. These include PfEMP2 (MESA), which interacts with the erythrocyte cytoskeletal protein 4.1^43–46^, and CLAG 3.1–3.2, which are proposed components of a nutrient channel in infected erythrocytes^47^.

Besides playing a role in egress, MSP1 is thought to mediate the merozoite’s interaction with the erythrocyte surface upon invasion^17,48–50^, after which time MSP1 and several other surface proteins are shed from the merozoite as it enters an erythrocyte^41^. This Ca^2+^-dependent protein shedding^51^, assayed as MSP-1 processing *in vitro* using purified merozoites, has apparent first-order kinetics with an MSP-1 processing half-life of approximately 20 minutes^52^. Here we show that FITC-heparin is also shed from live egressed merozoites in a similarly Ca^2+^-dependent manner. By not interfering with surface protein shedding, heparin might not inhibit merozoite penetration into erythrocytes. However, specific merozoite-erythrocyte interactions that precede invasion may be negatively affected by heparin. Indeed, several studies suggest that stages prior to merozoite penetration are blocked in the presence of heparin^17,22^.

The sudden appearance of a third heparin-binding site on the apical prominence of merozoites late after egress was independent of Ca^2+^-dependent protease activation, and seemed to occur with or without shedding of the second binding partner from parasite surfaces. Since the merozoite is fully formed at this third stage of apical heparin binding, the appearance of this late membrane-bound heparin target can occur either by merozoite extrusion or enzymatic modification of pre-existing surface proteins. Based on electron microscopy images of invading merozoites extruding rhoptry material into erythrocytes^37,38^ and observations that rhoptry proteins are well represented among the parasite’s heparin-binding proteins^36,53^, we suggest that this heparin target is a component of the extruded rhoptry material.

Our data suggest two heparin targets on the merozoite surface because merozoites could initiate a pseudo-invasion program under our experimental invasion-free conditions. In 1992, Trager *et al*.^54^ showed that extra-erythrocytic parasite development might occur *in vitro* in medium supplemented with erythrocyte homogenate. Here we show that regular culture medium supports early developmental steps. The dynamic interaction of heparin with merozoites that we observed using live-cell microscopy might explain a controversy originating from previous publications that used static-cell imaging. One paper described heparin binding to the apical region of merozoites^53^, while another showed heparin binding to the entire merozoite surface^55^. In addition, a developmental sequence of protein exposures regulated by a timed series of protein release from parasite secretory organelles is highly reminiscent of echinoderm zygotic development after fertilization^56^.

Conceptually, this work demonstrates that egress mechanisms can be targeted with large hydrophilic drugs, opening new avenues for antimalarial drug development that exploit the parasite’s own induction of a short-lasting stage-specific permeability to circumvent the anticipated membrane barrier to drug entry^57^. Heparin’s interaction with merozoite surface proteins and infection-modified erythrocyte membranes could be centrally important to understanding the critical mediators of parasite egress, parasite dispersion, and dissemination in general. Heparin’s fast entry is in part due to the very small volume between the merozoites and the plasma membrane just before egress, allowing diffusion to rapidly fill this space. Its action inside infected erythrocytes demonstrates the feasibility of employing proteins, peptides, or other natural polymers targeting the parasite egress mechanism to prevent parasite dissemination. Indeed, heparin recapitulates some of the effects exerted by immune antibodies^42^, and does so in several *P. falciparum* strains, thus lacking the specificity of antibodies. Polypeptides complementary to the functionally-conserved sequences of parasite proteins involved in parasite egress and invasion may be worth investigating to develop antimalarials that interact with intracellular parasites, including drug-resistant strains.

## Methods

### *P. falciparum* strains and human erythrocytes

We used three strains of *P. falciparum*: NF54, a long-term-adapted laboratory reference strain (ATCC, Manassas, VA); and CP803 and RF967, two short-term-adapted, artemisinin-resistant clinical isolates from Cambodian patients with malaria^58^. The clinical isolates were collected on a protocol (ClinicalTrials.gov identifier no. NCT00341003) approved by the Cambodian National Ethics Committee for Health Research and NIAID Institutional Review Board (IRB), in which patients gave written informed consent, and were maintained in culture in our laboratories.

### Generation of NF54 EXP2-mNeonGreen parasites

Integration of an mNeonGreen fusion at the endogenous EXP2 C-terminus was accomplished with CRISPR/Cas9 editing. A guide RNA target downstream of the *exp2* stop codon was chosen and the gRNA seed sequence was ordered as sense and antisense oligo pairs (P1/P2), annealed and inserted into the plasmid pAIO^59^ at BtgZI using an In-Fusion cloning kit (Clontech), resulting in the plasmid pAIO-EXP2-CT-gRNA. The vector pPM2GT^60^ was modified to replace the hDHFR selection cassette with a yDHODH selection cassette amplified from the plasmid pUF-1^61^ using primers P3/P4 and inserted between BglII and SalI sites, resulting in the vector pyPM2GT. A 5′ homology donor template (up to but not including the *exp2* stop codon) and a 3′ homology donor template (beginning 156 bp downstream of the *exp2* stop codon just after the gRNA seed sequence) were PCR amplified from *P. falciparum* NF54 genomic DNA (primers P5/6 and P7/8, respectively), assembled in a second PCR reaction using primers P7/P6, and inserted between XhoI and AvrII sites in pyPM2GT. The *mNeonGreen* coding sequence^62^ was amplified with primers P9/10 (adding a flexible linker between EXP2 and mNeonGreen) and inserted between AvrII and EagI sites, resulting in the plasmid pyPM2GT-EXP2-mNeonGreen. This vector was linearized at the AflII site between the 3′and 5′ donor sequences and co-transfected with pAIO-EXP2-CT-gRNA into *P. falciparum* NF54^attB^ parasites^63^. Selection with 2 µM DSM-1 was applied 24 hours post transfection. After returning from selection, integration at the intended site was confirmed by PCR using primers P11/P12. A clonal line containing the EXP2-mNeonGreen fusion was obtained by limiting dilution.

List of used primers for generation of NF54-EXP2-mNeonGreen parasites.

P1  TAAGTATATAATATTatattatgtacagtatctgaGTTTTAGAGCTAGAA

P2  TTCTAGCTCTAAAACtcagatactgtacataatatAATATTATATACTTA

P3  GGGAGACCGGCAGATCTTATAAGGAAATTCCC

P4  ATGCCTGCAGGTCGACTCTAGAGGATCCCCGG

P5  GTTTGATTATTTTATTTATGTACTCTCCTTATGACTTAAGCCTTGAGAGAAATATGGGAT

P6  CTGCACCTGGCCTAGGTTCTTTATTTTCATCTTTTTTTTCATTTTTAAATAAATCTCCAC

P7  CACTATAGAACTCGAGGGAGAAACAATCTTTTATATAAAATGTACAGAGTTTGAAAG

P8  ATCCCATATTTCTCTCAAGGCTTAAGTCATAAGGAGAGTACATAAATAAAATAATCAAAC

P9  GATGAAAATAAAGAACCTAGGGGAAGTGGAGGAGTGAGCAAGGGCGAGGAGGATAAC

P10  TAACTCGACGCGGCCGTCACTTGTACAGCTCGTCCATGCCCATC

P11  GCAACAAGTGCCTTAACCACCG

P12  GTAAGTCTTCTTCGACCTGCAC

*gRNA seed sequences are showing in lower case.

We maintained all parasite cultures in human erythrocytes isolated from the blood of healthy donors (who had consented to participate in the NIH IRB-approved Research Donor Program in Bethesda, MD; all samples were anonymized) in culture medium: RPMI 1640 medium supplemented with 25 mM HEPES, 0.1 mM hypoxanthine, 25 µg/ml gentamicin, 0.5% Albumax II (all from Gibco, Waltham, MA), and 4.5 mg/ml glucose (Sigma, St. Louis, MO). Cultures were maintained at low parasitemia and 5% hematocrit.

### Heparin

We used heparin (H3149, Sigma) at concentrations of 25–100 µg/ml in culture medium (~20 U/ml of physiological activity) or fluorescently-labelled heparin (FITC-heparin, H7482, Life Technologies, Waltham, MA) at concentrations of 1–20 µg/ml in culture medium.

### Inhibitors

Compound 2 (4-[7-[(dimethylamino)methyl]-2-(4-fluorphenyl) imidazo[1,2-a] pyridine-3-yl]pyrimidin-2-amine; MRT00072329) was provided by Dr. Simon Osborne, Medical Research Council Technology (MRCT), United Kingdom and E-64, a cysteine protease inhibitor, was purchased from Sigma (E3132).

### Parasite egress assays

We performed live-cell recording as described^24^ using an LSM 510 laser-scanning microscope (Carl Zeiss AG, Oberkochen, Germany) with a 63 × 1.4 NA oil objective and 488-nm laser with low light intensity to minimize cell photodamage. To observe parasite egress, we isolated schizonts from culture using 65% Percoll^64,65^, adjusted them to 0.1–0.2% hematocrit in media of different compositions at 37 °C, and placed them into special environmental chambers for microscopy (HybriWell HBW20, Grace Bio-Labs, Inc., Bend, OR). These chambers preserve cell viability for several hours, as well as egress sites, i.e., places where schizonts ruptured and released merozoites at the end of their IDC^24^. To quantify the effect of heparin on egress, we kept chambers at 37 °C for 60–90 minutes to accumulate egress sites, and then cooled them at 15 °C for 30 minutes to stop egress. We quantified egress as the fraction of schizonts releasing merozoites.

### Quantitative image analysis

We manually selected individual FITC-heparin-labeled merozoite regions of interest (ROIs, 2 μm × 2 μm) in time-lapse recordings to measure loss of cell fluorescence, excluding those that were tightly packed (<2 μm) or moving out of focus. We tracked relative merozoite positions using a cross-correlation search strategy, matching merozoites at each time point to a static reference template created by averaging the positions of four merozoites. This corrected for stage drift through time and for small lateral cell movements. We then measured the average fluorescence within each ROI, and time-centered this value at the initiation of fluorescence loss. We manually identified initiation points by detecting the end of the “shoulder” of fluorescence loss, and the beginning of the period resembling an exponential decay. We then fit single-exponential decay equations to the 500-second period beginning at the initiation point for each merozoite, and averaged the resulting time constants. We measured photobleaching by fitting exponential decay functions on a frame-wise basis during the photobleaching period at the end of experiments with blocked heparin complex shedding. We then multiplied the resulting frame constants by the time per frame to obtain a photobleaching time course.

To quantify the FITC-Heparin signal from the membrane of ghosts in single- and multiple-infected RBCs, one confocal image was taken at the equator of the RBC with a pinhole of 1 Airy Unit. All imaging parameters were kept constant after adjusting them to prevent detector saturation. RBC membrane fluorescence was then thresholded using the Otsu method^66^ to exclude background fluorescence. The mean pixel intensity of the thresholded image was taken as a data point.

All methods using in this research were carried out in accordance with relevant guidelines and regulations.

## Electronic supplementary material

Supplementary Movie 1

Supplementary Movie 2

Supplementary Movie 3

Supplementary Movie Legends

## References

[1] Miller LH, Ackerman HC, Su XZ, Wellems TE (2013). Malaria biology and disease pathogenesis: insights for new treatments. Nature medicine.

[2] Desai SA, Bezrukov SM, Zimmerberg J (2000). A voltage-dependent channel involved in nutrient uptake by red blood cells infected with the malaria parasite. Nature.

[3] Blackman MJ, Carruthers VB (2013). Recent insights into apicomplexan parasite egress provide new views to a kill. Current opinion in microbiology.

[4] Wickham ME, Culvenor JG, Cowman AF (2003). Selective inhibition of a two-step egress of malaria parasites from the host erythrocyte. The Journal of biological chemistry.

[5] Glushakova S, Yin D, Li T, Zimmerberg J (2005). Membrane transformation during malaria parasite release from human red blood cells. Current biology: CB.

[6] Abkarian M, Massiera G, Berry L, Roques M, Braun-Breton C (2011). A novel mechanism for egress of malarial parasites from red blood cells. Blood.

[7] Hale VL (2017). Parasitophorous vacuole poration precedes its rupture and rapid host erythrocyte cytoskeleton collapse in Plasmodium falciparum egress. Proceedings of the National Academy of Sciences of the United States of America.

[8] Chandramohanadas R (2009). Apicomplexan parasites co-opt host calpains to facilitate their escape from infected cells. Science.

[9] Das S (2015). Processing of Plasmodium falciparum Merozoite Surface Protein MSP1 Activates a Spectrin-Binding Function Enabling Parasite Egress from RBCs. Cell host & microbe.

[10] Glushakova S, Mazar J, Hohmann-Marriott MF, Hama E, Zimmerberg J (2009). Irreversible effect of cysteine protease inhibitors on the release of malaria parasites from infected erythrocytes. Cellular microbiology.

[11] Kafsack BF (2009). Rapid membrane disruption by a perforin-like protein facilitates parasite exit from host cells. Science.

[12] Glushakova S (2010). New stages in the program of malaria parasite egress imaged in normal and sickle erythrocytes. Current biology: CB.

[13] Deligianni E (2013). A perforin-like protein mediates disruption of the erythrocyte membrane during egress of Plasmodium berghei male gametocytes. Cellular microbiology.

[14] Wirth CC (2014). Perforin-like protein PPLP2 permeabilizes the red blood cell membrane during egress of Plasmodium falciparum gametocytes. Cellular microbiology.

[15] Garg S (2013). Calcium-dependent permeabilization of erythrocytes by a perforin-like protein during egress of malaria parasites. Nature communications.

[16] Yang AS (2017). Cell Traversal Activity Is Important for Plasmodium falciparum Liver Infection in Humanized Mice. Cell reports.

[17] Boyle MJ, Richards JS, Gilson PR, Chai W, Beeson JG (2010). Interactions with heparin-like molecules during erythrocyte invasion by Plasmodium falciparum merozoites. Blood.

[18] Leitgeb AM (2011). Low anticoagulant heparin disrupts Plasmodium falciparum rosettes in fresh clinical isolates. The American journal of tropical medicine and hygiene.

[19] Butcher GA, Parish CR, Cowden WB (1988). Inhibition of growth *in vitro* of Plasmodium falciparum by complex polysaccharides. Transactions of the Royal Society of Tropical Medicine and Hygiene.

[20] Vogt AM (2006). Release of sequestered malaria parasites upon injection of a glycosaminoglycan. PLoS pathogens.

[21] Aquino RS, Park PW (2016). Glycosaminoglycans and infection. Frontiers in bioscience.

[22] Crick AJ (2014). Quantitation of malaria parasite-erythrocyte cell-cell interactions using optical tweezers. Biophysical journal.

[23] Evans SG, Morrison D, Kaneko Y, Havlik I (1998). The effect of curdlan sulphate on development *in vitro* of Plasmodium falciparum. Transactions of the Royal Society of Tropical Medicine and Hygiene.

[24] Glushakova S, Yin D, Gartner N, Zimmerberg J (2007). Quantification of malaria parasite release from infected erythrocytes: inhibition by protein-free media. Malaria journal.

[25] Johnson D (1994). Characterization of membrane proteins exported from Plasmodium falciparum into the host erythrocyte. Parasitology.

[26] Haldar K, de Amorim AF, Cross GA (1989). Transport of fluorescent phospholipid analogues from the erythrocyte membrane to the parasite in Plasmodium falciparum-infected cells. The Journal of cell biology.

[27] Lauer SA, Rathod PK, Ghori N, Haldar K (1997). A membrane network for nutrient import in red cells infected with the malaria parasite. Science.

[28] Bubb MR, Senderowicz AM, Sausville EA, Duncan KL, Korn ED (1994). Jasplakinolide, a cytotoxic natural product, induces actin polymerization and competitively inhibits the binding of phalloidin to F-actin. The Journal of biological chemistry.

[29] Dvorin JD (2010). A plant-like kinase in Plasmodium falciparum regulates parasite egress from erythrocytes. Science.

[30] Collins CR, Hackett F, Atid J, Tan MSY, Blackman MJ (2017). The Plasmodium falciparum pseudoprotease SERA5 regulates the kinetics and efficiency of malaria parasite egress from host erythrocytes. PLoS pathogens.

[31] Beeson JG (2016). Merozoite surface proteins in red blood cell invasion, immunity and vaccines against malaria. FEMS microbiology reviews.

[32] Santos JM, Graindorge A, Soldati-Favre D (2012). New insights into parasite rhomboid proteases. Molecular and biochemical parasitology.

[33] Harris PK (2005). Molecular identification of a malaria merozoite surface sheddase. PLoS pathogens.

[34] Howell SA (2003). A single malaria merozoite serine protease mediates shedding of multiple surface proteins by juxtamembrane cleavage. The Journal of biological chemistry.

[35] Green JL, Hinds L, Grainger M, Knuepfer E, Holder AA (2006). Plasmodium thrombospondin related apical merozoite protein (PTRAMP) is shed from the surface of merozoites by PfSUB2 upon invasion of erythrocytes. Molecular and biochemical parasitology.

[36] Zhang Y (2013). Proteomic analysis of Plasmodium falciparum schizonts reveals heparin-binding merozoite proteins. Journal of proteome research.

[37] Miller LH, Aikawa M, Johnson JG, Shiroishi T (1979). Interaction between cytochalasin B-treated malarial parasites and erythrocytes. Attachment and junction formation. The Journal of experimental medicine.

[38] Riglar DT (2011). Super-resolution dissection of coordinated events during malaria parasite invasion of the human erythrocyte. Cell host & microbe.

[39] Wasserman M, Alarcon C, Mendoza PM (1982). Effects of Ca++depletion on the asexual cell cycle of Plasmodium falciparum. The American journal of tropical medicine and hygiene.

[40] Weiss GE (2015). Revealing the sequence and resulting cellular morphology of receptor-ligand interactions during Plasmodium falciparum invasion of erythrocytes. PLoS pathogens.

[41] Blackman MJ, Heidrich HG, Donachie S, McBride JS, Holder AA (1990). A single fragment of a malaria merozoite surface protein remains on the parasite during red cell invasion and is the target of invasion-inhibiting antibodies. The Journal of experimental medicine.

[42] Lyon JA, Thomas AW, Hall T, Chulay JD (1989). Specificities of antibodies that inhibit merozoite dispersal from malaria-infected erythrocytes. Molecular and biochemical parasitology.

[43] Magowan C (1995). Role of the Plasmodium falciparum mature-parasite-infected erythrocyte surface antigen (MESA/PfEMP-2) in malarial infection of erythrocytes. Blood.

[44] Waller KL (2003). Mature parasite-infected erythrocyte surface antigen (MESA) of Plasmodium falciparum binds to the 30-kDa domain of protein 4.1 in malaria-infected red blood cells. Blood.

[45] Enderle T (1997). Membrane specific mapping and colocalization of malarial and host skeletal proteins in the Plasmodium falciparum infected erythrocyte by dual-color near-field scanning optical microscopy. Proceedings of the National Academy of Sciences of the United States of America.

[46] Maier AG, Cooke BM, Cowman AF, Tilley L (2009). Malaria parasite proteins that remodel the host erythrocyte. Nature reviews. Microbiology.

[47] Nguitragool W (2011). Malaria parasite clag3 genes determine channel-mediated nutrient uptake by infected red blood cells. Cell.

[48] Su S, Sanadi AR, Ifon E, Davidson EA (1993). A monoclonal antibody capable of blocking the binding of Pf200 (MSA-1) to human erythrocytes and inhibiting the invasion of Plasmodium falciparum merozoites into human erythrocytes. Journal of immunology.

[49] Goel VK (2003). Band 3 is a host receptor binding merozoite surface protein 1 during the Plasmodium falciparum invasion of erythrocytes. Proceedings of the National Academy of Sciences of the United States of America.

[50] Baldwin MR, Li X, Hanada T, Liu SC, Chishti AH (2015). Merozoite surface protein 1 recognition of host glycophorin A mediates malaria parasite invasion of red blood cells. Blood.

[51] Blackman MJ, Holder AA (1992). Secondary processing of the Plasmodium falciparum merozoite surface protein-1 (MSP1) by a calcium-dependent membrane-bound serine protease: shedding of MSP133 as a noncovalently associated complex with other fragments of the MSP1. Molecular and biochemical parasitology.

[52] Blackman MJ, Chappel JA, Shai S, Holder AA (1993). A conserved parasite serine protease processes the Plasmodium falciparum merozoite surface protein-1. Molecular and biochemical parasitology.

[53] Kobayashi K (2013). Analyses of interactions between heparin and the apical surface proteins of Plasmodium falciparum. Scientific reports.

[54] Trager W, Williams J (1992). Extracellular (axenic) development *in vitro* of the erythrocytic cycle of Plasmodium falciparum. Proceedings of the National Academy of Sciences of the United States of America.

[55] Marques J (2014). Application of heparin as a dual agent with antimalarial and liposome targeting activities toward Plasmodium-infected red blood cells. Nanomedicine: nanotechnology, biology, and medicine.

[56] Matese JC, Black S, McClay DR (1997). Regulated exocytosis and sequential construction of the extracellular matrix surrounding the sea urchin zygote. Developmental biology.

[57] Sugano K (2010). Coexistence of passive and carrier-mediated processes in drug transport. Nature reviews. Drug discovery.

[58] Amaratunga C (2012). Artemisinin-resistant Plasmodium falciparum in Pursat province, western Cambodia: a parasite clearance rate study. The Lancet. Infectious diseases.

[59] Spillman, N. J., Beck, J. R., Ganesan, S. M., Niles, J. C. & Goldberg, D. E. The chaperonin TRiC forms an oligomeric complex in the malaria parasite cytosol. *Cellular microbiology* **19**, 10.1111/cmi.12719 (2017). 10.1111/cmi.12719 PMC5429184 28067475

[60] Klemba M, Beatty W, Gluzman I, Goldberg DE (2004). Trafficking of plasmepsin II to the food vacuole of the malaria parasite Plasmodium falciparum. The Journal of cell biology.

[61] Ganesan SM (2011). Yeast dihydroorotate dehydrogenase as a new selectable marker for Plasmodium falciparum transfection. Molecular and biochemical parasitology.

[62] Shaner NC (2013). A bright monomeric green fluorescent protein derived from Branchiostoma lanceolatum. Nature methods.

[63] Adjalley SH (2011). Quantitative assessment of Plasmodium falciparum sexual development reveals potent transmission-blocking activity by methylene blue. Proceedings of the National Academy of Sciences of the United States of America.

[64] Dluzewski AR, Ling IT, Rangachari K, Bates PA, Wilson RJ (1984). A simple method for isolating viable mature parasites of Plasmodium falciparum from cultures. Transactions of the Royal Society of Tropical Medicine and Hygiene.

[65] Lambros C, Vanderberg JP (1979). Synchronization of Plasmodium falciparum erythrocytic stages in culture. The Journal of parasitology.

[66] Otsu N (1979). Threshold Selection Method from Gray-Level Histograms. Ieee T Syst Man Cyb.

