# Supplementary material for: Exploitation of a newly-identified entry pathway into the malaria parasite-infected erythrocyte to inhibit parasite egress
Source: Sci Rep. 2017 Sep 25;7:12250. doi: 10.1038/s41598-017-12258-x (PMC5612957; doi:10.1038/s41598-017-12258-x)
Supplement: Supplementary file 4 — Supplementary Movie Legends [file 41598_2017_12258_MOESM4_ESM.doc]

**Exploitation of a newly-identified entry pathway into the malaria parasite-infected erythrocyte to inhibit parasite egress**

Svetlana Glushakova, Brad L. Busse, Matthias Garten, Josh R. Beck,

Rick M. Fairhurst, Daniel E. Goldberg & Joshua Zimmerberg

**Supplementary movie legends**

**Supplementary Movie 1. Heparin inhibits parasite egress in vitro.** *P. falciparum* NF54 schizonts were exposed to 100 µg/mL of heparin and imaged using laser-scanning confocal microscopy. DIC microscopy images are shown. Note that there are different intervals of time between frames.

**Supplementary Movie 2. Heparin enters the infected erythrocyte just before erythrocyte membrane rupture.** *P. falciparum* CP803 schizonts were exposed to 20 µg/mL of FITC-heparin and imaged using laser-scanning confocal microscopy. Split DIC and fluorescence microscopy images of a double-infected erythrocyte are shown. The interval of time between heparin influx into the erythrocyte and parasite egress is 12-16 seconds.

**Supplementary Movie 3. A different heparin target appears on the merozoite surface after heparin is shed from the merozoite surface.** *P. falciparum* NF54 schizonts were exposed to 10 µg/mL of FITC-heparin and imaged using laser-scanning confocal microscopy. Merged DIC and fluorescence microscopy images are shown. Note that heparin binds material extruded from the apical prominence of merozoites.
